# An Immunoproteomic Survey of the Antibody Landscape: Insights and Opportunities Revealed by Serological Repertoire Profiling

**DOI:** 10.3389/fimmu.2022.832533

**Published:** 2022-02-01

**Authors:** Steven Ionov, Jiwon Lee

**Affiliations:** Thayer School of Engineering, Dartmouth College, Hanover, NH, United States

**Keywords:** Ig-Seq, serological antibody repertoire, proteomics, infectious disease, cancer, autoimmunity

## Abstract

Immunoproteomics has emerged as a versatile tool for analyzing the antibody repertoire in various disease contexts. Until recently, characterization of antibody molecules in biological fluids was limited to bulk serology, which identifies clinically relevant features of polyclonal antibody responses. The past decade, however, has seen the rise of mass-spectrometry-enabled proteomics methods that have allowed profiling of the antibody response at the molecular level, with the disease-specific serological repertoire elucidated in unprecedented detail. In this review, we present an up-to-date survey of insights into the disease-specific immunological repertoire by examining how quantitative proteomics-based approaches have shed light on the humoral immune response to infection and vaccination in pathogenic illnesses, the molecular basis of autoimmune disease, and the tumor-specific repertoire in cancer. We address limitations of this technology with a focus on emerging potential solutions and discuss the promise of high-resolution immunoproteomics in therapeutic discovery and novel vaccine design.

## Introduction

The discovery of a substance in serum with the ability to protect against disease dates back to Emil von Behring and Shibasaburo Kitasato (1); just a year later, Paul Ehrlich made the first reference to ‘*Antikörper’*, or antibodies, in an 1891 report (2). No less important in retrospect was Karl Landsteiner’s discovery 50 years later that antisera contain specificities to multiple antigens (3); this may be viewed as the discovery of an antibody *repertoire*. The serological repertoire is comprised of a diverse combination of immunoglobulins secreted by B cells in various compartments including peripheral blood, bone marrow, and mucosal sites (4, 5). From initial exposures to exogenous and endogenous (in the case of cancer and autoimmune disease) antigens, the antibody repertoire is established and constantly reshaped through subsequent exposures to a multitude of different antigens during one’s lifetime (6, 7).

Characterization of serum antibodies has traditionally relied on serological assays that determine the total abundance, binding specificity, and neutralizing activity of polyclonal antibodies using various techniques, including enzyme-linked immunosorbent assays (ELISA) as well as neutralization and immunofluorescence assays (8, 9). Though serology remains essential in the present-day study of antibody responses (10) and gives critical insights into the global immune repertoire, it does not inform on the traits of individual constituent antibody molecules. More recently, single B cell sequencing has allowed recombinant expression and characterization of monoclonal antibodies (mAbs), leading to functional delineation of antibody responses at the single-mAb level and discovery of numerous mAbs with potent therapeutic activity (11). However, as some B cells do not secrete antibodies, B-cell-based studies are often unable to accurately determine the relative abundance of each identified mAb, or its relevance to serological protection. As protective antibody molecules that circulate in blood or coat mucosal surfaces are the key correlate of humoral immunity to various diseases (12), proteomic studies of abundant immunoglobulins are critical to in-depth analysis of the antibody landscape.

**Figure 1:**
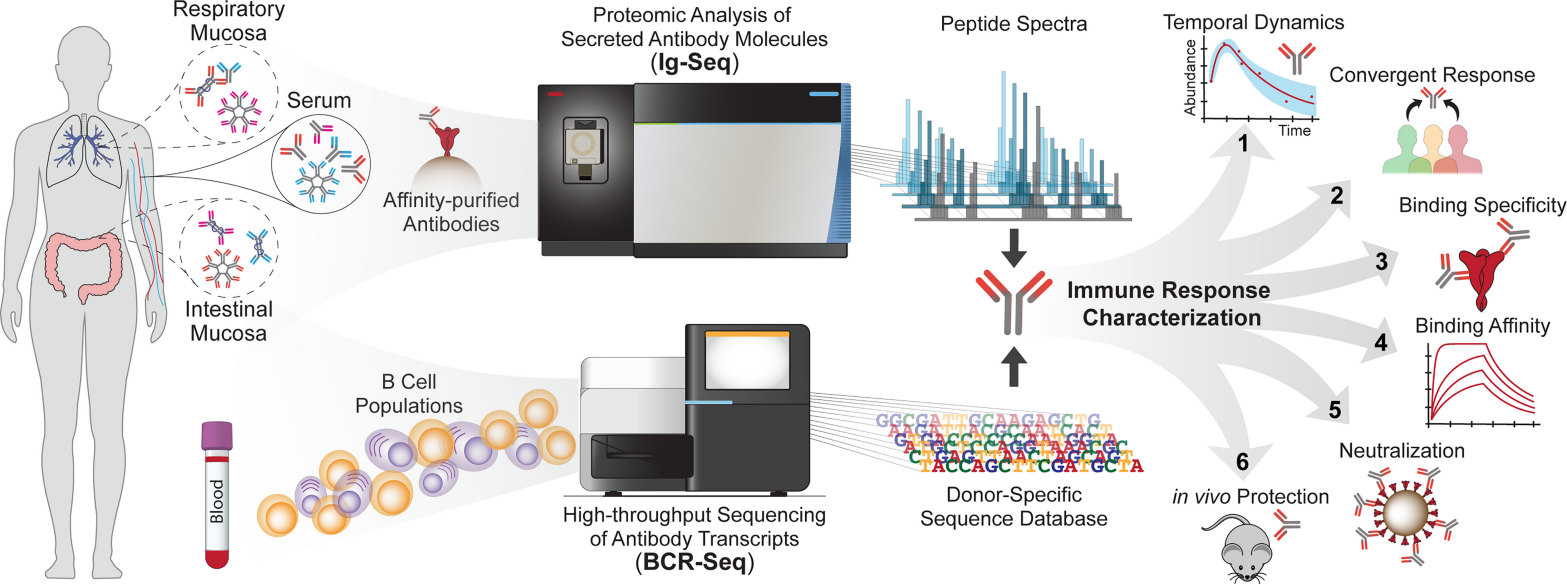
Quantitative and qualitative profiling of antibody repertoires using the Ig-Seq pipeline. Antibodies sampled from biological fluids are subject to affinity purification against an antigen of interest and profiled by mass spectrometry (top pathway). A donor-specific reference database generated from BCR-Seq (bottom pathway) is used to match peptide spectra with antibody sequences. Relative antibody abundances are profiled in detail and can be tracked longitudinally. Ig-Seq enables detailed longitudinal profiling of antibody repertoires (1), identification of convergent responses (2), functional characterization of antibody specificity (3) and affinity (4), as well as delineation of the *in vitro* neutralization (5) and *in vivo* protection (6) conferred by abundant antibodies. Insights from the repertoire’s behavior after antigenic exposure, and the protective features of expressed mAbs, inform the design of diagnostics, vaccination strategies, and therapeutics.

Over the last two decades, mass spectrometry (MS) has found increasing use in the analysis of complex protein samples (13); more recently, it has been applied to profiling of polyclonal antibody mixtures, giving rise to next-generation serology (14–17). The proteomic deconvolution of antigen-specific serum antibody mixtures, pioneered by the Georgiou group, is known as Ig-Seq (14, 15, 18) (**Figure 1**). This method has allowed identification, quantification, and longitudinal tracking of antibody lineages at the molecular level. Ig-Seq is a bottom-up proteomic approach involving affinity purification of antibodies against a target antigen, followed by analysis *via* a liquid-chromatography-tandem-MS (LC-MS/MS) system. Generated peptide spectra are then matched to a sequence database (19), often constructed by high-throughput B cell sequencing [BCR-Seq, reviewed in (20–22)] to identify serum antibodies and enable their subsequent recombinant expression as mAbs for further functional study (15, 23–25). Technical advances and challenges in mass-spectrometry based antibody sequencing, from sample preparation to computational pipelines, have been recently reviewed comprehensively by Greiff and colleagues (26). In this mini review, we present a survey of the various pathologies explored to date using antibody mass spectrometry, highlighting unique insights into the characteristics of the disease-specific immune repertoire and the therapeutic molecules or strategies which may arise from these studies. We emphasize the implications of disease-specific insights in combating infectious disease, autoimmunity, and cancer.

## Applications of Ig-Seq to Study Disease

### Infectious Disease

#### Influenza

In 1947, a seminal study observed that university students infected with influenza, who had been previously vaccinated against a different influenza strain, had higher serum antibody titers against the original vaccine strain than the infecting strain (27); this serologic phenomenon was later described by the authors as ‘original antigenic sin’ (28). Numerous studies have since demonstrated that the antibody repertoire generated from early exposure to influenza is ‘imprinted’ on the immune system. This set of antibodies persists in circulation and exerts a major influence on the nature of the antibody response upon subsequent exposure (27, 29–33). Despite mounting evidence of immune imprinting in the context of influenza, precise understanding of how these pre-existing antibodies can influence the elicitation of new antibodies has been impeded by the inability to identify pre-existing antibodies using bulk serological assays. Lee et al. addressed this gap by using Ig-Seq to quantitatively interrogate the serological repertoire of young adults before and after seasonal influenza vaccination to differentiate between pre-existing (*i.e.*, already present in circulation before the vaccination) and *de novo* (*i.e.*, newly elicited following the vaccination) repertoires (34). The authors showed that >60% of the post-vaccination hemagglutinin (HA)-specific repertoire consisted of pre-existing antibodies which tended to be cross-reactive to H1 and H3 subtypes while *de novo* antibodies were mostly specific to one subtype, suggesting that the pre-existing population targeted conserved regions on HA. Recombinant expression of representative cross-reactive antibodies revealed a conserved, previously uncharacterized epitope present at the ‘interface’ of trimeric HA. These antibodies conferred prophylactic and therapeutic protection in mice against divergent influenza strains. Other groups have since confirmed the protective ability and prevalence of HA interface-targeting antibodies (35–38).

A subsequent study investigated the longitudinal dynamics of the HA-reactive antibody repertoire in an individual across 5 years of repeated exposure to the same H1N1 (A/California/4/2009; CA09) strain (39). With Ig-Seq enabling the identification and quantification of influenza-specific serum antibodies at multiple timepoints, the authors showed that a small group of ‘persistent’ antibodies circulating in serum across five years made up ~70% of the CA09-reactive serum titer, demonstrating the remarkable stability of serological antibody repertoires. In other words, each vaccination elicited new antibodies making up ~30% of the anti-HA titer, but these ‘transient’ antibodies decayed away while the ‘imprinted’ repertoire remained. The persistent antibodies were more mutated and displayed more cross-reactivity to a divergent H5N1 influenza strain compared to non-persistent antibodies.

Most of the population is exposed to influenza at an early age, whether due to infection by the circulating virus or through childhood vaccines (40). Older adults are likely to have been exposed on many occasions to both viral and vaccine antigens. The ability to elicit *de novo* protective immune responses decreases with old age (41), accentuating the importance of a broadly protective persistent repertoire. This impact of age on the influenza-reactive antibody repertoire has been investigated by determining the relative abundances of antibodies cross-reactive to H1 and H3 across adults aged 26-70 before and after seasonal vaccination (42). The study revealed that older individuals (suggesting increased exposure events) had a larger relative abundance of cross-reactive antibodies, with over 90% of the vaccine-specific repertoire in some elderly donors displaying reactivity to both H1 and H3. Strikingly, subsequent in-depth characterization of broadly cross-reactive serum antibodies revealed that they recognized sulfated glycans abundant in avian egg-prepared vaccines, likely rendering them ineffective in preventing infection. Collectively, the quantitative nature of Ig-Seq and the ability to track antibodies longitudinally enabled studies focusing on the molecular and functional features of imprinted influenza serum antibody repertoires.

#### SARS-CoV-2

The onset of the COVID-19 pandemic raised the urgency to understand the immune response to viral infection, and more recently, vaccination. Serological studies of SARS-CoV-2-infected patients and analysis of their B cells led to rapid profiling of longitudinal antibody responses to infection (43–46), contributing to the unprecedented speed of new and effective vaccine development (47) and discovery of neutralizing mAbs (48–53). While these data have improved our understanding of protection afforded by serum antibodies, the relative abundance and functionalities of the individual SARS-CoV-2-reactive antibodies circulating in blood have remained unknown. The traits of individual antibodies are clinically important based on previous serological repertoire analyses, which indicate a high degree of polarization [*i.e.*, a small number of antibodies comprising a large fraction of the overall response (14, 15, 34, 39, 42, 54)]. To address this, Ippolito and colleagues profiled the SARS-CoV-2 Spike (S) protein-reactive serological repertoire in COVID-19 patients during early convalescence (55). They determined that over 80% of S-specific IgG in sera bound to regions other than the receptor-binding domain (RBD), a primary target of neutralizing antibodies. Instead, many highly abundant antibodies (some of which individually comprised >20% of the S-specific repertoire) bound to the N-terminal domain (NTD), and functional characterization of those NTD-binding mAbs showed robust neutralization activity (IC_50_ as low as 10 ng/mL). However, NTD binding decreased or was completely ablated when the authors introduced mutations present in SARS-CoV-2 variants of concern (56). Thus, a sizable portion of antibodies were demonstrably susceptible to mutations in their binding epitopes, implying that their protective ability was not robust against emerging mutants.

Given that anti-S IgG binding titers are thought to be well-correlated with protection against SARS-CoV-2 infection (10), a recent study sought to determine the longitudinal dynamics of individual S-reactive serum antibodies over four months in convalescent patients (57). The authors specifically tracked peptides containing the complementarity determining region of heavy chain 3 (CDR-H3) using multiple reaction monitoring (MRM) (58), which enables precise quantitation of pre-selected peptides within a complex mixture. The authors built a model simulating the decay of S-specific serum antibodies and determined that antibody levels drop below a chosen seroconversion threshold of 1:40 around 70 days after onset of symptoms. This finding is in agreement with the observation of short-lived nature of antibody responses following SARS-CoV-2 infections (59), although the thresholds for protection are as yet uncertain. This work further highlights the urgency to better understand the longevity of SARS-CoV-2-specific antibodies in secretions, which may present important implications for developing vaccines capable of eliciting durable antibody responses.

#### Other Infectious Disease

The therapeutic impact of broadly-neutralizing antibodies (bnAbs) has been demonstrated in infectious disease (60), suggesting that the antibody repertoire can be an expansive reservoir for mining potent therapeutic candidates against microbial pathogens. Lindesmith et al. longitudinally profiled patients before and after vaccination against norovirus (HuNoV) (54), and found a pre-existing antibody, A1431, that was boosted 126-fold by vaccination. A1431 bound to conserved viral capsid epitopes and neutralized a broad panel of HuNoV strains, including strains first observed years after the patient was vaccinated. The sera of patients with chronic infections such as HIV have also been studied with similar methods, as chronic stimulation of B cells by viruses that undergo immune escape may give rise to bnAbs (61). For example, one study functionally characterized abundant antibodies against the HIV Env glycoprotein MPER region in a chronically infected donor (62). The authors swapped the chains of several bnAbs found using Ig-Seq to generate a chimeric mAb that neutralized 206/208 global isolates of HIV-1. Similar work has been done to analyze antibodies identified in sera collected from 2 HIV elite-neutralizers (63), finding one in particular, N49P7, which was able to neutralize 117/117 pseudoviruses of a global panel due to its ability to bind conserved inner-layer residues of the gp120 glycoprotein (64). Additionally, Ig-Seq-based repertoire profiling has been similarly applied in malaria (65) and hepatitis C (66), leading to the isolation and delineation of mAbs with protective qualities.

### Autoimmune Disease

While the secreted antibody repertoire may act as the principal protective force in infectious disease immunity, the same repertoire acts as a primary agent of pathogenesis in autoimmune conditions (67–69). To this end, groups have applied Ig-Seq to study serologic signatures of systemic lupus erythematosus (SLE), namely anti-dsDNA antibodies and antibodies expressing the 9G4 idiotype (‘9G4 antibodies’) (70, 71). One study sequenced anti-dsDNA antibodies from SLE patients and found a preference for mutations to arginine in antibody variable regions, consistent with observations from previous B cell sequencing studies that these mutations enable DNA binding (71). Others have compared spectra of purified 9G4 antibodies with sequences of donor-matched antibody-secreting cells (ASC’s), which expand rapidly during SLE-associated disease ‘flares’ (70). The sequences from ASC’s matched peptide spectra exactly, indicating that these ASC’s, which were derived from naïve B cells, had a profound impact on the SLE serological repertoire. In the case of celiac disease patients, a more recent study found a gene preference for *IGHV5-51* in plasma cells reactive to TG2, a primary celiac antigen (72). The researchers quantified the percentage of *IGHV5-51* antibodies in anti-TG2 IgA repertoires of patients in various stages of disease and observed that *IGHV5-51* percentage was well correlated with disease severity, suggesting a pathogenic role for these antibodies. Looking forward, proteomic study of the ‘autoantibody repertoire’ with Ig-Seq may help us understand this repertoire’s behavior before symptom onset, a crucial step in the reversal or treatment of autoimmunity (70, 72–74).

In some cases, trends observed in B cell populations and the secreted repertoire differ, highlighting the utility of direct proteomic analysis in characterizing the mechanisms of autoimmune pathology. In an illustrative example, Stanley and colleagues followed up on an earlier study (75) investigating B cells specific to the Dsg3 autoantigen in pemphigus vulgaris patients, interrogating the anti-Dsg3 autoantibodies in serum using Ig-Seq (76). Their initial B cell sequencing work had revealed a gene preference for *IGHV1-46* among the Dsg3-reactive B cell population, but the new study demonstrated that the functional, secreted antibody repertoire did not share this preference, and was more diverse than what was observed in the B cell compartment. Moreover, longitudinal analysis over several years revealed a substantial change in the abundances of individual anti-Dsg3 antibodies in circulation over time, though a subset of the highly abundant antibodies persist for years, as well as a significant portion of antibody sequences observed by transcriptomic methods.

### Cancer

Though few studies have examined cancer antibody repertoire *via* Ig-Seq, initial studies indicate that MS-based analysis of sera can be used for increased sensitivity in monitoring malignancies. For example, a study investigating multiple myeloma (MM) patient serum antibodies (77) was able to detect MM-specific antibodies in patients that had returned false-negative results *via* protein electrophoresis and serum immunofixation, both standard procedures for molecular detection of myeloma (78). A separate study (79) attempted to create a screen for non-small-cell lung cancer based on serum antibody CDR’s detected using mass spectrometry. This work suggests that peptide signatures have potential as tools for cancer detection (80), though other studies in humans and animal models have suggested that antigen-specific antibody repertoires do not exhibit major overlap between individuals (76, 81, 82). Separately, interrogating the tumor immune repertoire has resulted in the identification of tumor-specific antibodies, which can be developed as therapeutics or used to discover novel binding targets (83). McDaniel et al. (84) analyzed serum antibodies from breast cancer patients who had tested positive for the cancer testis antigen NY-ESO-1, a marker normally confined to male germ cells but present in up to 25% of breast cancers. Serum antibodies identified from Ig-Seq and expressed as mAbs bound to NY-ESO-1 with K_D_s as low as 2.0 nM.

## Alternative Methods for Immunoproteomic Analysis of Disease

Ig-Seq is a bottom-up proteomic method involving injection of proteolytically-digested peptides into the LC-MS system (85); trypsin is a good choice for Ig-Seq studies due to the prevalence of arginine or lysine residues flanking the antibody CDR-H3 region. However, proteolytic digestion with trypsin or any other proteases may lead to loss of detectable CDR-H3 peptides and reduce the quantity of identifiable CDR-H3 sequences. To address this, other groups have used top-down or middle-down proteomic approaches, which preserve sequence coverage at the expense of resolution; nevertheless, these methods have resulted in useful insight regarding the antibody repertoire [reviewed in (26)]. Bottom-up approaches have also sought to increase coverage by digesting samples with complementary proteases and using computational pipelines to construct intact antibody sequences (86). Separately, Ig-Seq relies on reference antibody databases to deconvolute peptide spectra; the quality, size and source of the database can significantly impact the results and accuracy of antibody identification (26). Several groups are attempting to overcome this dependence using germline gene sequence databases available through online resources, such as IMGT (72, 87, 88). However, database matching cannot discern exact sequences of antibodies, as their CDR-H3’s specifically are formed only after somatic hypermutation in their secreting B cells. Reference-free sequencing of antigen-specific immunoglobulins from biological fluids *via* mass spectrometry would address this limitation, though this remains difficult due to the complexity and high variability of antibody variable regions. Recent methods have enabled sequencing of full-length purified mAbs at close to 100% accuracy (89), implementing error correction with database homology searches and introducing mutations and post-translational modifications to converge on exact sequences. Some groups have combined similar pipelines with database matching and have been successful in sequencing antibodies from highly restricted populations, as in autoimmune disease (90). More recent work suggests that antibodies will soon be sequenced directly from repertoires with enough precision to allow recombinant expression (82, 91).

## Discussion

Numerous studies have utilized Ig-Seq to identify individual antibodies in serum and track how their abundances change over time in the contexts of various pathologies (34, 39, 42, 54, 55, 57, 66, 76). This is of particular interest in viral diseases, as there is increasing evidence that exposure history plays a decisive role in the antibody response to infection or vaccination (39, 54, 92, 93). Though the effects of immune imprinting have been most extensively studied in influenza (34, 39, 42), recent work in HuNoV has revealed boosting of persistent cross-reactive antibodies in response to vaccination (54). Further, emerging research in SARS-CoV-2 indicates that the antibody repertoire may be shaped by previous infection with endemic coronaviruses, suggesting that exposure history may play a critical role in other viral immune responses (92, 93). Tracking individual antibody abundances over longitudinal samples will allow us to measure the relative contributions to protection made by pre-existing and *de novo* antibodies. Describing both of these antibody populations on a disease-specific basis will be necessary for understanding the immunopathogenesis of infection and evaluating the effectiveness of vaccines.

The Ig-Seq pipeline has yielded high-affinity, broadly neutralizing antibodies reactive to SARS-CoV-2, influenza, HIV, and HuNoV, demonstrating the broad potential of the method in discovering novel therapeutics (54, 62, 64). Notably, this method has allowed groups to identify immune ‘signatures’ of exposure, such as the presence of broadly-reactive antibodies against select influenza (34) or norovirus strains (54), or non-protective antibodies to a conserved epitope on SARS-CoV-2 (93). These and similar data may inform the design of next-generation vaccines customized for an individual’s exposure history. For example, a novel vaccine may present epitopes known to boost pre-existing, broadly neutralizing antibodies and avoid presenting epitopes known to be associated with previous non-protective immune responses. It is worth noting, however, that this strategy cannot predict immune escape mutations, which may occur even on conserved epitopes.

Ig-Seq holds promise as a means to profile the secreted antibody repertoire at mucosal surfaces, including the respiratory and intestinal tracts (94–98) (**Figure 1**). Functional characterizations of site-specific antibody repertoires in diseases occurring at mucosal surfaces are highly important for treatment of individuals with respiratory conditions, such as cystic fibrosis and lung cancer. In addition, novel vaccines are being developed to target mucous membranes (99), and quantitative profiling of the mucosal immune repertoire will be essential to understand their effectiveness and function. As the source of the antibody transcript sequence database influences the quality of Ig-Seq data, subsequent work will require generating more comprehensive donor-specific reference databases using B cells from various compartments. Alternatively, substantial advances in reference-free sequencing may soon enable identification of serum antibodies without the need for such databases.

While mass spectrometric studies of the antibody repertoire span a wide breadth of disease types, more depth of study is needed to obtain additional data for developing novel therapeutic strategies in each disease case, and to validate the protective effects of those strategies. Ig-Seq will allow us to interrogate the overall immune response in unprecedented detail and use site-specific characteristics of the antibody repertoire to design novel therapeutics and vaccine strategies.

## Author Contributions

SI and JL wrote and revised the manuscript. All authors contributed to the article and approved the submitted version.

## Funding

This work was supported by the National Institutes of Health [P20GM113132] and the Cystic Fibrosis Foundation [STANTO19R0].

## Conflict of Interest

The authors declare that the research was conducted in the absence of any commercial or financial relationships that could be construed as a potential conflict of interest.

## Publisher’s Note

All claims expressed in this article are solely those of the authors and do not necessarily represent those of their affiliated organizations, or those of the publisher, the editors and the reviewers. Any product that may be evaluated in this article, or claim that may be made by its manufacturer, is not guaranteed or endorsed by the publisher.
